# Metabolomic Profiling of Gestational Diabetes Mellitus and Its Subtypes Among Chinese Pregnant Women

**DOI:** 10.3390/life16101641

**Published:** 2026-09-30

**Authors:** Xialidan Alifu, Yiwen Qiu, Boya Wang, Nuo Xu, Shuhui Wang, Haoyue Cheng, Ye Huang, Libi Zhang, Diliyaer Ainiwan, Haibo Zhou, Hui Liu, Danqing Chen, Yunxian Yu

**Affiliations:** 1 School of Basic Medicine, Xinjiang Key Laboratory of Molecular Biology for Endemic Diseases, Xinjiang Medical University, Urumqi 830017, China; 2 Department of Epidemiology & Health Statistics, School of Public Health, School of Medicine, Zhejiang University, Hangzhou 310058, China; 3 Department of Public Health, and Department of Anesthesiology, Second Affiliated Hospital of Zhejiang University School of Medicine, Hangzhou 310009, China; 4 Clinical Research Center, Sir Run Run Shaw Hospital, School of Medicine, Zhejiang University, Hangzhou 310058, China; 5 Women’s Hospital, School of Medicine, Zhejiang University, Hangzhou 310006, China

**Keywords:** gestational diabetes mellitus, heterogeneous disease, thyroid function, untargeted metabolomics

## Abstract

**Background:** This study aimed to explore first-trimester metabolic profiles associated with gestational diabetes mellitus (GDM) and its glycemic subtypes. **Methods:** This nested case–control study included 90 women with GDM, comprising 30 with isolated fasting hyperglycemia (IFH), 30 with isolated post-load hyperglycemia (IPH), and 30 with combined hyperglycemia (CH), together with 30 women with normal glucose tolerance (NGT). Untargeted UHPLC-MS metabolomic profiling was performed on fasting plasma samples collected during the first trimester. Exploratory metabolic features were compared between GDM or its subtypes and NGT, and Spearman correlation analyses were performed to examine associations between candidate metabolic features and maternal fasting blood glucose (FBG), BMI, thyroid-related parameters, parity, education, and gestational weight gain. Exploratory pathway analyses were conducted to characterize potentially related metabolic processes. **Results:** At the exploratory threshold of VIP > 1 and nominal *p* < 0.05, 107 candidate metabolic features were observed in the GDM vs. NGT comparison, with 89, 76, and 123 candidate features observed in the IFH vs. NGT, IPH vs. NGT, and CH vs. NGT comparisons, respectively. These candidate features were mainly annotated as organoheterocyclic compounds and lipids and lipid-like molecules, with differences in chemical-class distributions across subtype comparisons. Organosulfur-related annotations were observed among the IFH candidate features, whereas alkaloid-related annotations were observed among the CH candidate features. No individual feature remained statistically significant after multiple-testing correction. Correlation analyses identified FDR-adjusted associations between selected candidate metabolic features and maternal FBG, BMI, and thyroid-related parameters. Exploratory pathway mapping highlighted processes related to lipid, amino acid, carbohydrate, and nucleotide metabolism. **Conclusions:** This exploratory study suggests that first-trimester plasma metabolic patterns may differ among women who subsequently develop different glycemic subtypes of GDM. The observed candidate features, clinical correlations, and pathway-level patterns provide hypotheses regarding metabolic heterogeneity in GDM but should not be considered validated biomarkers or subtype-specific metabolic signatures. Further studies using targeted metabolomics and larger independent cohorts are needed to confirm these observations.

## 1. Background

Gestational Diabetes Mellitus (GDM) is a condition characterized by normal sugar metabolism before pregnancy, with the onset of diabetes occurring during pregnancy. GDM raises the risk of adverse outcomes for both the mother and the fetus, in both the short (e.g., preterm birth, macrosomia) [1] and long term (e.g., type 2 diabetes and cardiovascular diseases) [2], posing a significant threat to maternal and fetal health. According to the 11th edition of the International Diabetes Federation Diabetes Atlas, approximately 15.6% of live births worldwide were affected by GDM in 2024, with the corresponding estimate for China being approximately 15.7% [3]. Our previous study indicates that the incidence rate in the coastal areas of Zhejiang Province was as high as 21.6% [4]. Although dietary and physical activity interventions are common clinical methods to reduce the risk of GDM, their precise implementation is challenging and often does not achieve the desired clinical effect.

Fasting and postprandial hyperglycemia may reflect partly distinct pathophysiological abnormalities. Fasting glucose is strongly influenced by hepatic glucose production and hepatic insulin sensitivity, whereas postprandial glucose regulation additionally depends on glucose-stimulated insulin secretion and peripheral glucose uptake, particularly in skeletal muscle [5,6,7,8,9]. IFH has been associated predominantly with hepatic insulin resistance and impaired early-phase insulin secretion, whereas IPH has been linked more closely to impaired insulin secretion and skeletal-muscle insulin resistance with relatively preserved hepatic insulin sensitivity [9]. Thus, abnormalities in fasting versus postprandial glucose may represent different metabolic phenotypes rather than interchangeable manifestations of GDM. Monnier et al. [10] reported that the relative contributions of fasting and postprandial hyperglycemia differed across the progression of diabetes. However, the respective roles of fasting and postprandial glucose abnormalities in overall glycemic regulation remain incompletely understood [11]. Accordingly, we classified GDM into three glycemic subtypes based on the pattern of abnormal glucose values during the oral glucose tolerance test (OGTT): isolated fasting hyperglycemia (IFH), isolated postprandial hyperglycemia (IPH), and combined hyperglycemia (CH) [4]. CH may reflect a combination of the abnormalities observed in both IFH and IPH. These physiological differences suggest that GDM is metabolically heterogeneous and that analyzing GDM as a single entity may obscure subtype-specific metabolic alterations.

Metabolomics enables comprehensive profiling of small-molecule metabolites and has increasingly been used to investigate metabolic disturbances associated with GDM [12,13]. In GDM, the most frequently disrupted metabolic pathways involve amino acids (such as glutathione, alanine, valine, and serine), carbohydrates (including 2-hydroxybutyrate and 1,5-anhydroglucitol), and lipids (specifically phosphatidylcholines and lysophosphatidylcholines) [14,15,16,17]. Previous studies have reported alterations in amino acid, carbohydrate, and lipid metabolism in women with GDM. However, most metabolomic studies have treated GDM as a single disease entity and have not examined whether metabolic alterations differ according to fasting and postprandial glycemic phenotypes. Whether distinct GDM glycemic subtypes are preceded by different metabolic profiles in early pregnancy therefore remains unclear.

To address this gap, we conducted a nested case–control study within the Zhoushan Pregnant Women Cohort. Plasma samples collected during the first trimester (8–13 weeks of gestation), before the diagnosis of GDM, were analyzed using untargeted metabolomics. GDM was subsequently diagnosed by a 75-g OGTT at 24–28 weeks of gestation and classified as IFH, IPH, or CH according to the pattern of abnormal glucose values. We aimed to (1) characterize first-trimester metabolomic features associated with subsequent GDM and its three glycemic subtypes; (2) explore associations between selected metabolomic features and maternal clinical characteristics. These analyses were intended to characterize early metabolic heterogeneity across GDM subtypes and generate hypotheses regarding their underlying pathophysiology.

## 2. Materials and Methods

### 2.1. Study Participants

This nested case–control study was based on the ongoing Zhoushan Pregnant Women Cohort (ZPWC) Study, conducted in Zhoushan Maternal and Child Care Hospital in Zhoushan, Zhejiang province, China, since August 2011. In the ZPWC study, the inclusion criteria of pregnant women were as follows: (1) consented to participate in the study; (2) enrolled between the 8th and 12th week of gestation; (3) completed prenatal examinations and gave birth at the Zhoushan Maternal and Child Care Hospital; (4) age between 18 and 45 years; and (5) no family history of mental illness. The exclusion criteria included: (1) a history of severe chronic or acute diseases; (2) any history of mental disorders before pregnancy; (3) threatened miscarriage; (4) fetal malformations or abnormal fetal development; and (5) inability to complete the questionnaire due to intellectual issues. The detailed criteria have been described in a previous study based on this cohort [4].

In this manuscript, the pregnant women needed to meet the additional following conditions: (1) underwent a thyroid function test once in first trimester; (2) had an OGTT once in the second trimester; (3) had no thyroid disease or take related medications before or during pregnancy; (4) had no history of chronic diabetes before pregnancy; and (5) singleton pregnancy.

**Figure 1:**
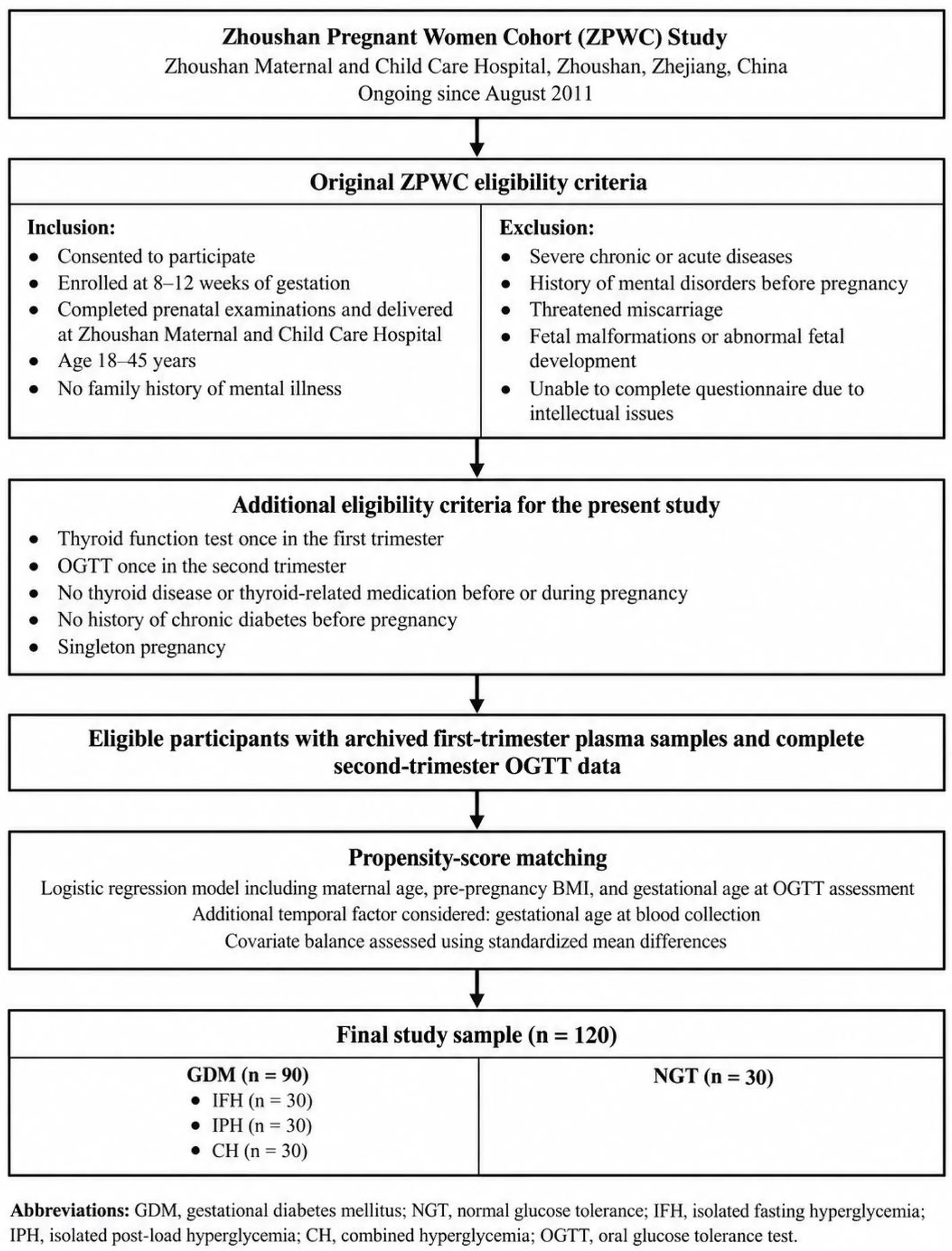
Flow diagram for recruitment using case–control studies.

Propensity scores for GDM were estimated using a logistic regression model including maternal age, pre-pregnancy BMI, and gestational age at the OGTT assessment. Although matching was performed according to gestational age at OGTT, metabolomic measurements were obtained from first-trimester samples. Gestational age at blood collection was additionally considered as an important temporal factor. Eligible participants were selected using propensity-score matching, resulting in 120 participants, including 90 women with GDM (30 with IFH, 30 with IPH, and 30 with CH) and 30 women with NGT. Covariate balance before and after matching was assessed using standardized men differences. The sample size was determined by the availability of eligible participants with archived first-trimester plasma samples and complete second-trimester OGTT data. No a priori sample size or power calculation was performed; therefore, the present study was considered exploratory and hypothesis-generating. A flow diagram for recruitment using case–control studies is presented in Figure 1.

### 2.2. Socio-Demographic Information and Blood Sample Collection

After enrollment, face-to-face interviews were conducted to collect information on socio-demographics. Details of their last menstrual cycle, conception method, parity, family history of diabetes, previous history of GDM, pre-pregnancy height, and weight were collected using a structured questionnaire during the first and second trimesters. Gestational age was initially determined from the last menstrual period and later validated through ultrasound imaging. Fasting blood samples were collected during the first trimester (8–12 weeks of gestation) after an overnight fast of at least 8 h. Blood collection was performed in the morning, and participants were generally advised to attend for sampling before 09:00, although the exact collection time varied according to their arrival at the hospital. Blood samples were centrifuged on the day of collection, after which plasma was separated and stored at −80 °C until metabolomic analysis. Blood samples were centrifuged, then the plasma and white blood cells were separated and frozen at −80 °C. The archived plasma samples used in the present study had been collected within the preceding five years; therefore, storage duration varied among participants but did not exceed approximately five years. BMI was calculated as the weight in kilograms divided by the square of height in meters.

### 2.3. Measurement of OGTT and Definition of GDM and GDM Subtypes

OGTT was performed between 24 and 28 weeks of gestation. Participants were instructed to fast for at least 8 h overnight and then consume a solution containing 75 g of glucose dissolved in 300 mL of water within 5 min the following morning. Venous blood samples were taken at three time points: before OGTT (0 h), 1 h and 2 h after OGTT, to measure the levels of plasma glucose. All OGTT measurements were performed in the clinical laboratory of Zhoushan Maternal and Child Care Hospital using the same standardized procedure. Plasma glucose levels were immediately measured using the hexokinase method with commercially available kits on a Beckman AU5800 automated biochemical analyzer (Beckman Coulter Inc., Brea, CA, USA). The OGTT results are retrieved from the Electronic Medical Records system. GDM diagnosis is made according to the International Association of Diabetes and Pregnancy Study Groups (IADPSG) criteria (met any one of the following conditions): fasting plasma glucose (FBG) of 5.1 mmol/L or higher; 1-h post-load glucose (PG1H) of 10 mmol/L or higher; or 2-h post-load glucose (PG2H) of 8.5 mmol/L or higher [18].

GDM subtypes were defined as follows: IFH, with isolated FBG ≥ 5.1 mmol/L; IPH, with isolated PG1H ≥ 10 mmol/L and/or PG2H ≥ 8.5 mmol/L; and CH, with both increased FBG (≥5.1 mmol/L) and post-load plasma glucose (PG1H ≥ 10 mmol/L and/or PG2H ≥ 8.5 mmol/L).

### 2.4. Untargeted Metabolomics Processing

A 100 μL aliquot of plasma was extracted with 400 μL of a solvent mixture (MeOH:ACN, 1:1, *v*/*v*) containing a proprietary mixture of isotopically labeled internal standards. Six internal-standard signals (IS1–IS6) were monitored for analytical quality control and internal-standard normalization; the exact chemical identities were not disclosed by the analytical service provider. The mixture was vortexed for 30 s, sonicated for 10 min in a 4 °C water bath, and incubated at −40 °C for 1 h to precipitate proteins. Samples were then centrifuged at 12,000 rpm (13,800× *g*) for 15 min at 4 °C, and the supernatant was transferred to a fresh glass vial for analysis.

A pooled quality-control (QC) sample was prepared by mixing equal aliquots of the supernatants from the experimental samples [19]. Fourteen pooled QC injections were included in the original analytical sequence to monitor analytical stability. QC performance was evaluated using total-ion chromatograms, PCA-based QC clustering, correlations among QC samples, and the response stability of the six isotopically labeled internal standards (IS1–IS6). The QC samples showed high pairwise correlations, exceeding the reference criterion of 0.85. The relative standard deviations (RSDs) of the six internal-standard signals ranged from 0.82% to 2.42%, with a median of approximately 1.87%, below the prespecified reference criterion of 10%. Blank samples were interspersed throughout the analytical sequence to monitor background signals and potential carryover.

LC–MS/MS analysis was performed using a Vanquish UHPLC system (Thermo Fisher Scientific, Waltham, MA, USA) equipped with a Waters ACQUITY UPLC BEH Amide column (2.1 mm × 50 mm, 1.7 μm) coupled to an Orbitrap Exploris 120 mass spectrometer (Thermo Fisher Scientific, Waltham, MA, USA). The mobile phase consisted of 25 mmol/L ammonium acetate and 25 mmol/L ammonium hydroxide in water (pH 9.75) as mobile phase A and acetonitrile as mobile phase B. The autosampler temperature was maintained at 4 °C, and the injection volume was 2 μL. The mass spectrometric conditions were as follows: sheath gas flow rate, 50 Arb; auxiliary gas flow rate, 15 Arb; capillary temperature, 320 °C; full-MS resolution, 60,000; MS/MS resolution, 15,000; stepped normalized collision energies, 20/30/40; and spray voltage, 3.8 kV in positive-ion mode and −3.4 kV in negative-ion mode.

The raw LC–MS data were converted to mzXML format using ProteoWizard, version 3 (ProteoWizard Software Foundation), and subsequently processed using an in-house R-based workflow incorporating XCMS for feature detection, extraction, alignment, and integration [20].

Metabolite identification confidence was assessed according to the Metabolomics Standards Initiative (MSI) framework. Level 1 identification was assigned when the MS1, MS2, and retention time (RT) of a detected metabolite matched those of an authentic reference standard. Level 2 annotation was assigned when both MS1 and MS2 spectra matched those available in public spectral databases. Level 3 annotations were based on matching with theoretical databases using MS1, MS2, and predicted RT, whereas Level 4 represented unknown features. In the present study, only metabolites classified as MSI Level 1 or Level 2 were retained for downstream metabolite-level analyses and biological interpretation. Level 1 compounds were considered confirmed metabolites, whereas Level 2 compounds were regarded as putatively annotated metabolites.

### 2.5. Variable Definition

Thyroid hormones were routinely tested during pregnancy and the data on thyroid function tests were extracted from the biochemical databases. Qualified nurses collected fasting venous blood samples from pregnant mothers in the first trimester, and the concentration of thyroid hormones including TSH, FT3, and FT4 was subsequently measured by Beckman Coulter UniCel Dxl 800 Access Immunoassay analyzer and kit in the laboratory of Zhoushan Maternal and Child Care Hospital. We utilized three indices, the Thyroid feedback quantile-based index (TFQI), thyrotropin index (TSHI), and thyrotroph thyroxine resistance index (TT4RI), to evaluate central sensitivity to thyroid hormones. Positive results for thyroid hormones were indicated by anti-thyroid peroxidase autoantibody (TPOAb) concentrations >34.0 IU/mL and anti-thyroglobulin antibodies (TgAb) concentrations >115 IU/mL, respectively. Pre-pregnancy BMI was calculated as weight in kilograms divided by height in meters squared and divided into four categories: underweight (<18.5 kg/m^2^); normal (18.5~23.9 kg/m^2^); overweight (24.0~27.9 kg/m^2^); obesity (≥28 kg/m^2^). Information on active smoking and alcohol consumption was obtained through routine medical history collection at the first antenatal examination. The categorical demographic variable was defined as “Unknown” if there was no response.

### 2.6. Statistical Analysis

A total of 25,488 metabolic features were initially extracted from the raw LC–MS data. Following preprocessing and quality filtering, 22,612 features were retained for statistical analysis, corresponding to the removal of 2876 features (11.28% of the initially extracted features). Preprocessing included RSD/CV-based filtering and missingness filtering. Features with >50% missingness were excluded, and the remaining missing values were imputed using one-half of the minimum observed value. Because the archived preprocessing report did not provide separate counts for features removed by the missingness and RSD/CV criteria, the number removed specifically because of >50% missingness could not be determined. Similarly, the exact number and proportion of individual values subsequently imputed were not available. Internal-standard normalization was subsequently performed.

Unsupervised principal component analysis (PCA) was performed to visualize the overall distribution of the samples and to identify potential outliers. Orthogonal partial least-squares discriminant analysis (OPLS-DA) was additionally performed as an exploratory visualization approach to assess group-related metabolic patterns. Seven-fold cross-validation and 200 permutation tests were used to examine model stability and potential overfitting. Given the limited sample size and the low cross-validated Q^2^ values, OPLS-DA was not considered evidence of predictive discrimination. Variable importance in projection (VIP) values were therefore used only as an exploratory measure of feature contribution and not as an inferential criterion for statistical significance.

For univariate comparisons, metabolic features were compared between the GDM and NGT groups and between each GDM subtype and the NGT group using independent-samples tests. To account for multiple comparisons in the high-dimensional metabolomic dataset, nominal *p* values were adjusted separately within each comparison using the Benjamini–Hochberg false discovery rate (FDR) procedure. An FDR-adjusted *p* value (*q* value) < 0.05 was considered statistically significant.

No individual metabolic feature remained statistically significant after FDR correction in any of the primary comparisons. Therefore, features meeting the original exploratory criteria of nominal *p* < 0.05 and VIP > 1 were retained only for descriptive and hypothesis-generating analyses and are referred to throughout the manuscript as exploratory candidate metabolic features or nominally differential metabolic features, rather than statistically significant differentially abundant metabolites.

Spearman correlation analyses were performed to explore the associations between exploratory candidate metabolic features and maternal clinical characteristics, including first-trimester fasting blood glucose (FBG), BMI, thyroid function parameters, gestational weight gain, education, and parity. To account for multiple correlation tests, the corresponding *p* values were adjusted using the Benjamini–Hochberg false discovery rate (FDR) procedure. Correlations with an absolute Spearman correlation coefficient (|r|) > 0.30 and an FDR-adjusted *p* value (*q* value) < 0.05 were reported. Given the exploratory feature-selection procedure and the limited sample size, these correlations were interpreted as exploratory associations rather than evidence of causal or mechanistic relationships.

Clinical characteristics were compared between groups using appropriate tests according to variable type and distribution. Correlations between candidate metabolic features and maternal clinical characteristics were assessed using Spearman correlation analysis. Where multiple correlation tests were performed, *p* values were adjusted using the Benjamini–Hochberg FDR procedure. Pathway enrichment analyses were conducted as exploratory, hypothesis-generating analyses. The reported pathway enrichment *p* values were nominal and were not adjusted for multiple pathway testing. Accordingly, these analyses were not used to establish statistically significant pathway-level associations. Pathways with nominal *p* < 0.05 were retained only for descriptive and hypothesis-generating interpretation, particularly given that the pathway analyses were based on exploratory candidate metabolic features and that several pathways contained only a small number of mapped features. All statistical analyses were performed using R software (Version 4.1.2; R Foundation for Statistical Computing, Austria) and SIMCA 18.0.1 where appropriate.

## 3. Results

### 3.1. Study Populations

Baseline characteristics revealed that there were significant differences in first trimester FT4, FT3/FT4 ratio, and weight gain from first to second trimester observed between total GDM (Table 1a), three GDM subtypes (Table 1b) and non-GDM groups, respectively. No significant differences in age, BMI, education level, parity, blood pressure, alcohol drinking and tobacco smoking were observed between these four groups.

### 3.2. Multivariate Statistical Analysis of Plasma Metabolites of GDM Subgroups and NGT

Overall, 22,612 features were included in the statistical analyses after data preprocessing and cleanup. Unsupervised PCA was used to assess the sample’s gross distribution and stability of the analysis process. The sample distribution in the first two highest principal components accounted for 21% of the variance in LC-MS (Figure S1). Initially, we examined the entire samples of 120 women to obtain an overview of the difference in the first trimester metabolites between GDM and NGT women. OPLS-DA was used only as an exploratory visualization of group-related metabolic patterns. In the GDM vs. NGT comparison, the model yielded R^2^X = 0.073, R^2^Y = 0.716, and cross-validated Q^2^ = 0.071 (Figure S2A). The low Q^2^ value did not support reliable discrimination between groups. The corresponding permutation analysis yielded Q^2^ = −0.47 (Figure S2B).

**Figure 2:**
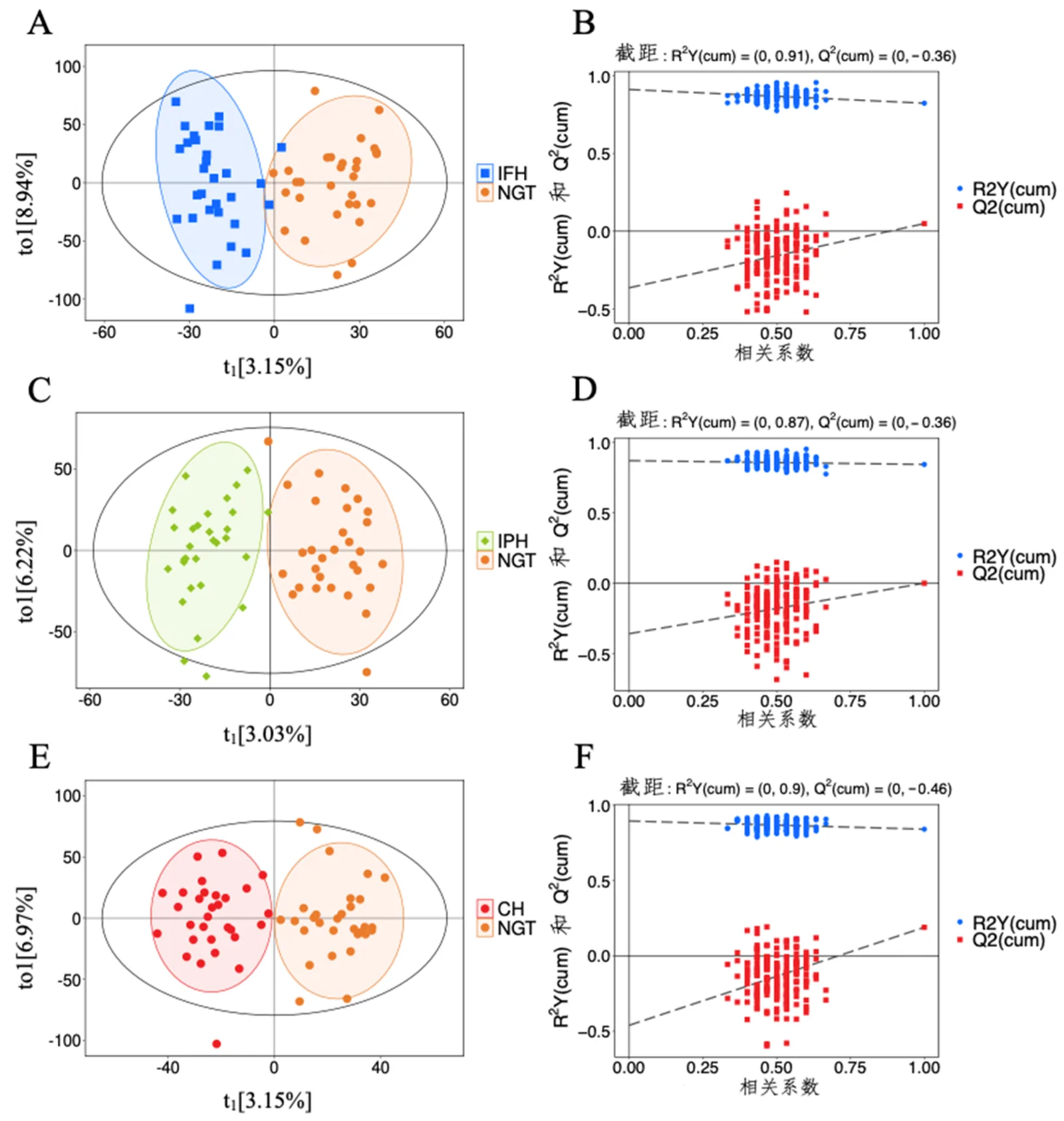
Exploratory OPLS-DA visualization of metabolic profiles across GDM subtype comparisons. (**A**,**C**,**E**): OPLS-DA model of IFH vs. NGT, IPH vs. NGT, and CH vs. NGT groups, respectively. (**B**,**D**,**F**): permutation test of IFH vs. NGT, IPH vs. NGT, and CH vs. NGT groups, respectively. R^2^Y describes model fit to the class-response matrix, whereas Q^2^ represents cross-validated model performance. The low Q^2^ values observed in the present analyses did not support reliable discrimination between groups. In the permutation-test plots, the dashed lines represent the regression lines for R_2_Y and Q_2_, respectively, whereas the solid horizontal line indicates the zero reference level.

Similarly, pairwise OPLS-DA score plots were generated for the three GDM subtype comparisons (Figure 2A,C,E). The R^2^X, R^2^Y, and cross-validated Q^2^ values were 0.121, 0.823, and 0.049 for IFH vs. NGT; 0.095, 0.843, and 0.001 for IPH vs. NGT; and 0.104, 0.841, and 0.189 for CH vs. NGT, respectively. Permutation analyses yielded Q^2^ values of −0.36, −0.36, and −0.46, respectively (Figure 2B,D,F). Collectively, these low cross-validated Q^2^ values did not support reliable discrimination between groups. Accordingly, OPLS-DA was interpreted only as an exploratory visualization, and VIP values were used solely for exploratory feature ranking.

### 3.3. Exploratory Metabolic Features Associated with GDM and Its Glycemic Subtypes

After correction for multiple testing using the Benjamini–Hochberg false discovery rate (FDR) procedure, no individual metabolic feature remained statistically significant at q < 0.05 in the GDM vs. NGT, IFH vs. NGT, IPH vs. NGT, or CH vs. NGT comparisons. Therefore, no feature-level association was considered statistically significant after multiple-testing correction. For exploratory and hypothesis-generating purposes, we further examined metabolic features meeting the nominal criteria of *p* < 0.05 and VIP > 1. These features are hereafter referred to as exploratory candidate metabolic features rather than statistically significant differentially abundant metabolites.

**Figure 3:**
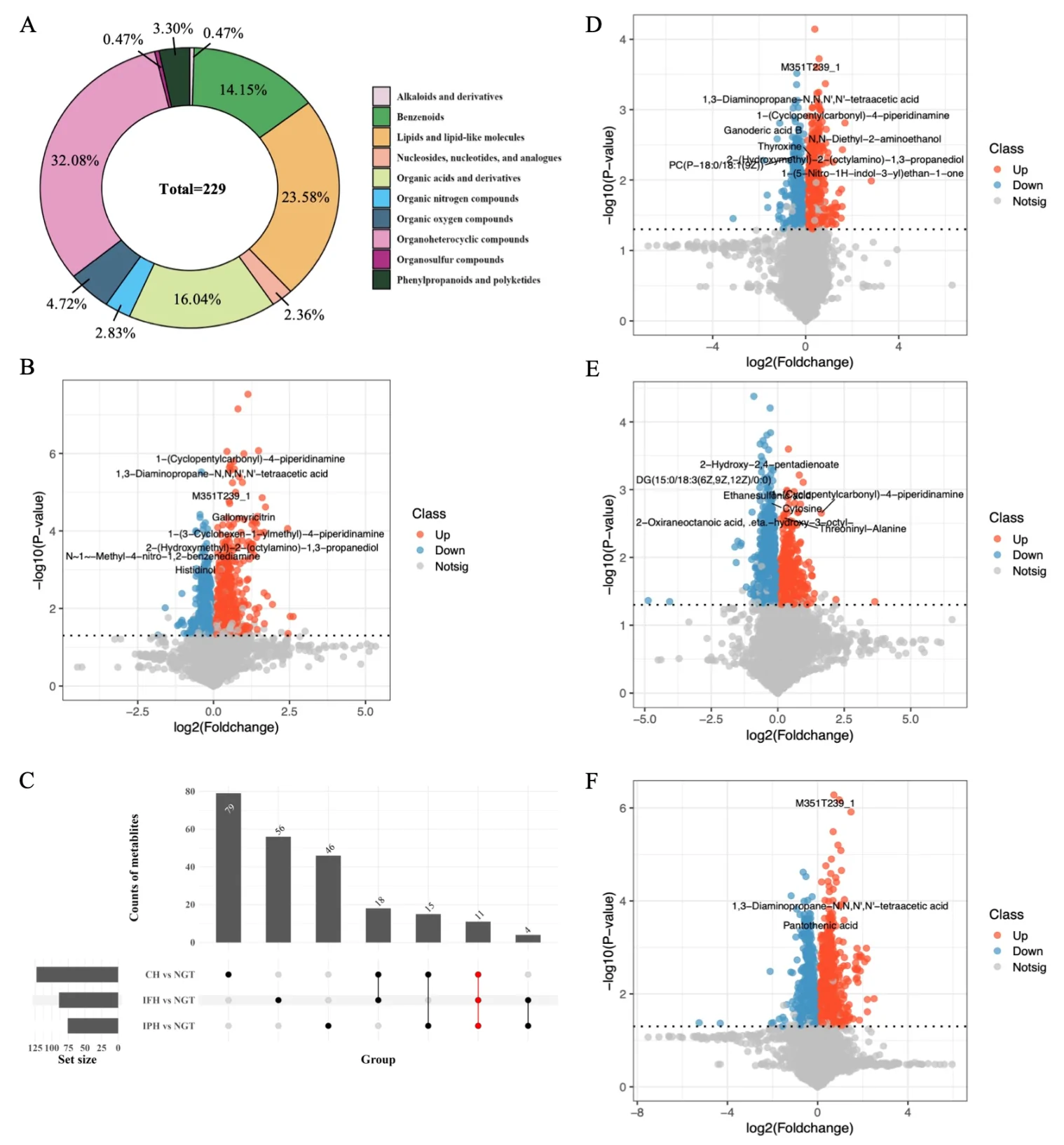
Exploratory analysis of candidate metabolic features associated with GDM and its glycemic subtypes. (**A**) Distribution of chemical classes among exploratory candidate metabolic features. (**B**) Volcano plot for total GDM vs. NGT. (**C**) UpSet plot showing overlapping candidate features across the IFH vs. NGT, IPH vs. NGT, and CH vs. NGT comparisons. Colored dots and connecting lines indicate the comparison sets included in each intersection, while the vertical bars represent the number of candidate features in each intersection. (**D**–**F**) Volcano plots for IFH vs. NGT, IPH vs. NGT, and CH vs. NGT, respectively. Highlighted features met the exploratory criteria of nominal *p* < 0.05 and VIP > 1. Labels indicate selected candidate metabolic features and have been repositioned to minimize overlap and improve readability. No individual metabolic feature remained statistically significant after Benjamini–Hochberg FDR correction at *q* < 0.05. Therefore, highlighted features should be interpreted as exploratory candidates rather than statistically confirmed differential metabolites.

Among the exploratory candidate features in the overall GDM vs. NGT comparison, organoheterocyclic compounds (32.08%) and lipids and lipid-like molecules (23.5%) represented the two largest chemical classes (Figure 3A). Using the exploratory criteria of nominal *p* < 0.05 and VIP > 1, 107 candidate metabolic features were observed in the comparison between total GDM and NGT groups (Figure 3B and Table S1). These features did not survive FDR correction and therefore should not be interpreted as statistically confirmed differences between the groups.

In the subtype analyses, 89 exploratory candidate features were observed in the IFH vs. NGT comparison (Figure 3D and Table S2), 76 in the IPH vs. NGT comparison (Figure 3E and Table S3), and 123 in the CH vs. NGT comparison (Figure 3F and Table S4). Exploratory examination of the chemical-class distributions suggested some differences across subtype comparisons. In particular, organosulfur-related features were observed among the candidate features in the IFH vs. NGT comparison, whereas alkaloids and derivatives were observed among those in the CH vs. NGT comparison. Other candidate features were distributed across several chemical classes, including benzenoids and lipids and lipid-like molecules (Figure S3). Because none of the individual features remained significant after FDR correction, these apparent subtype-related chemical-class patterns should be regarded as descriptive observations rather than evidence of confirmed subtype-specific metabolic alterations.

The UpSet plot further showed that 11 exploratory features were shared across all three subtype comparisons (Figure 3C). These included features putatively annotated as uric acid, glycolate, (3R)-3-hydroxy-L-proline, 1,3-diaminopropane-N,N,N′,N′-tetraacetic acid, 1-(cyclopentylcarbonyl)-4-piperidinamine, DL-norvaline, nodakenitin, N,N′-di(1,3,4-thiadiazol-2-yl)imidoformamide, carbamoyl phosphate, and 2-hydroxy-2,4-pentadienoate, together with the feature M351T239_1 (M351T239_1 remained an unannotated metabolic feature and was therefore not included in metabolite-level biological interpretation). Given that the same NGT reference group was used for the three subtype comparisons, these overlapping features do not represent independent replication. In addition, because most metabolite identities were based on database annotation and none of these features survived FDR correction, the overlap should be interpreted as exploratory and hypothesis-generating.

### 3.4. Exploratory Associations Between Candidate Metabolic Features and Maternal Clinical Characteristics

**Figure 4:**
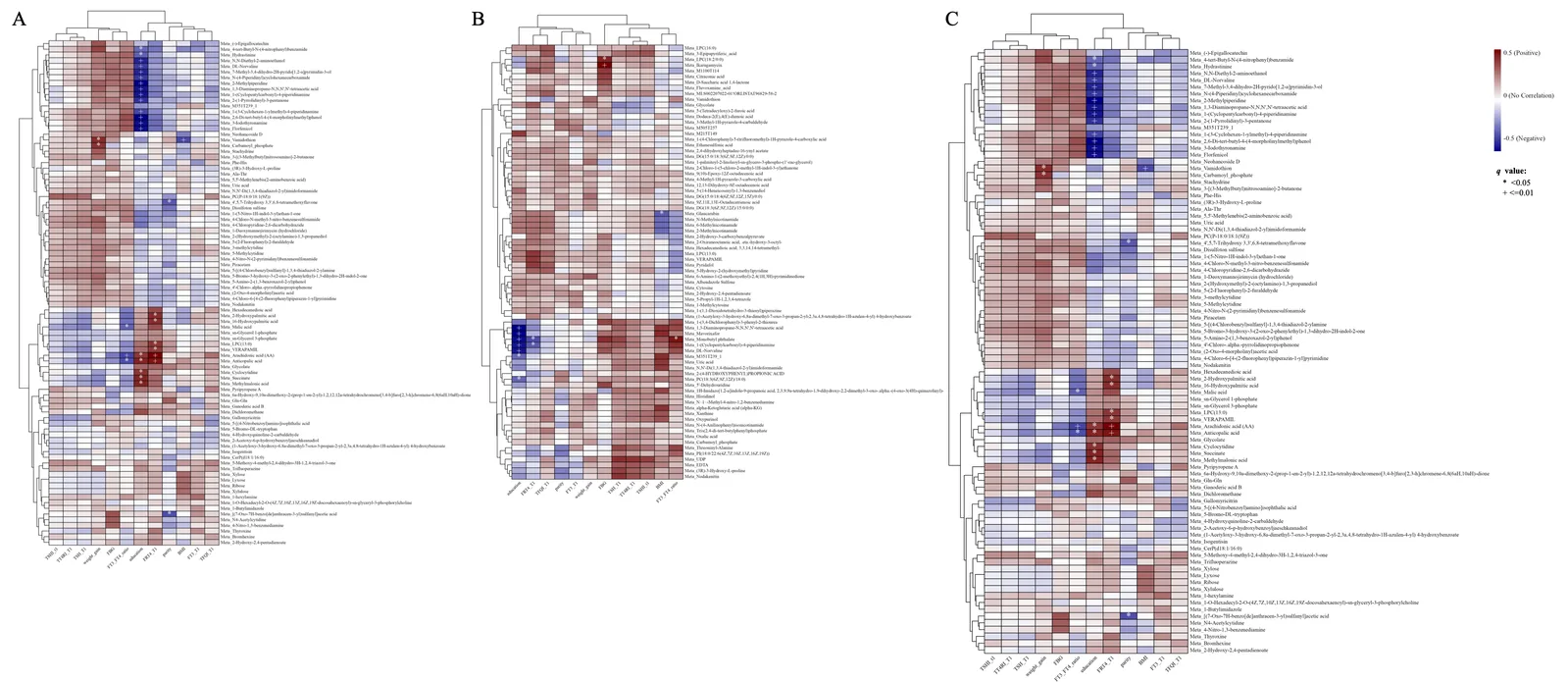
Heatmaps illustrating the clustering and correlations of annotated exploratory candidate metabolic features in IFH vs. NGT (**A**), IPH vs. NGT (**B**), and CH vs. NGT groups (**C**) with maternal plasma glucose concentrations (FBG, fasting blood glucose in the first trimester), maternal BMI, early pregnancy thyroid hormones, weight gain from first trimester to the second trimester, education, and parity. Corrected *p* values (*q* values) < 0.05 were considered significant. The level of significance is indicated with differently shaped symbols, * for *q* < 0.05 and + for *q* ≤ 0.01. Abbreviations: BMI, body mass index; FRT4_T1, free thyroxine in first trimester.

Given the nominal metabolic signals observed in the GDM subtype comparisons, we further explored their associations with maternal clinical characteristics, including first-trimester FBG, BMI, thyroid function parameters, gestational weight gain, education, and parity. Spearman correlation analyses were performed between the exploratory candidate metabolic features and these maternal characteristics, followed by correction for multiple testing. The heat map in Figure 4 presents a visual representation of the correlations between above mentioned first trimester indicators with altered metabolites across three GDM subtype groups and control groups. Highlighting the differences in the intensity and distribution of different metabolites. Higher values (red) indicate increased metabolite abundance, whereas lower values (blue) indicate decreased abundance. The results of the correlation analysis are in the Supplementary Materials (Tables S5–S7). We have identified the following correlations, characterized by a correlation coefficient > 0.3 and corrected *p* values (*q* values) < 0.05 (Table 2).

(1) The correlations between the indicators with altered metabolites across IFH and NGT: ① Maternal education positively correlated with anticopalic acid, arachidonic acid (AA), cyclocytidine, methylmalonic acid and succinate; negatively correlated with hydrastinine, etc.; ② FRT4 positively correlated with 2-Hydroxypalmitic acid, anticopalic acid, arachidonic acid (AA); ③ FT3/FT4 ratio negatively correlated with anticopalic acid, arachidonic acid (AA), malic acid; ④ maternal BMI negatively correlated with vamidothion; ⑤ maternal weight gain positively correlated with carbamoyl phosphate and vamidothion.
(2) The correlations between the indicators with altered metabolites across IPH and NGT: ① Maternal education negatively correlated with 1-(Cyclopentylcarbonyl)-4-piperidinamine, 1,3-Diaminopropane-N,N,N′,N′-tetraacetic acid, DL-Norvaline, mavorixafor, monobutyl phthalate, PC(18:3(6Z,9Z,12Z)/18:0); ② FRT4 negatively correlated with 1-(Cyclopentylcarbonyl)-4-piperidinamine, monobutyl phthalate; ③ FT3/FT4 ratio positively correlated with monobutyl phthalate.
(3) The correlations between the indicators with altered metabolites across CH and NGT: ① Education negatively correlated with DL-Norvaline, florfenicol, monobutyl phthalate, nicotine, etc.; ② maternal BMI positively correlated with 2-Aminonicotinic acid, 4-nitroaniline, nicotinamide N-oxide, Threonine, etc.; ③ FBG positively correlated with fradiamine B (also known as 3-[5-[Acetyl(hydroxy) amino] pentylcarbamoyl]-5-[3[acetyl(hydroxy) amino] propylamino]-3-hydroxy-5-oxopentanoic acid); ④ FRT4 negatively correlated with 1-(Cyclopentylcarbonyl)-4-piperidinamine, monobutyl phthalate; ⑤ FT3/FT4 ratio positively correlated with 3-Iodothyronamine, 4-Nitroaniline, 6-Deoxyfagomine, DL-Norvaline, florfenicol, glucuronic acid, monobutyl phthalate, nicotine, PC(38:3), etc.

### 3.5. Exploratory Pathway Analysis of Candidate Metabolic Features

**Figure 5:**
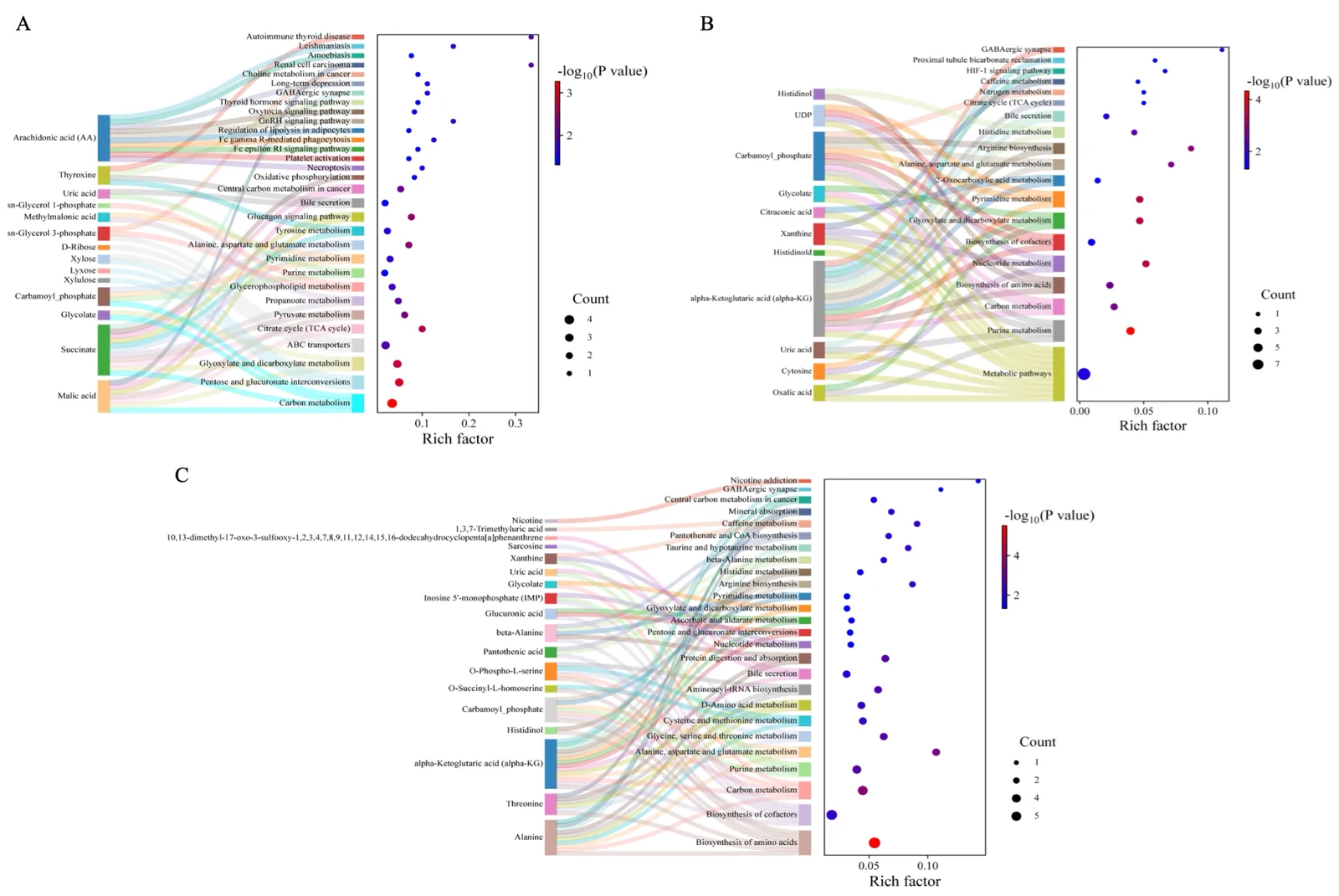
Exploratory KEGG pathway analyses. (**A**–**C**) Nominal pathway-level enrichment patterns in the IFH vs. NGT, IPH vs. NGT, and CH vs. NGT comparisons, respectively. Sankey diagrams show candidate metabolic features and their mapped pathways. In the bubble charts, point color represents the nominal enrichment *p* value, point size represents the number of mapped candidate features, and the rich factor represents the proportion of mapped features in each pathway. *p* values were not adjusted for multiple pathway testing; therefore, the displayed pathways should be interpreted as exploratory and hypothesis-generating rather than statistically significant pathway-level associations.

Exploratory KEGG pathway enrichment analyses were performed to characterize the biological processes to which the candidate metabolic features mapped. The reported enrichment *p* values were nominal and were not adjusted for multiple pathway testing; therefore, pathways with nominal *p* < 0.05 were interpreted only as exploratory pathway-level signals rather than statistically significant pathway-level associations. In the GDM vs. NGT comparison, candidate metabolic features mapped to pathways including general metabolic pathways, biosynthesis of cofactors, carbon metabolism, biosynthesis of amino acids, and purine metabolism (Table S8). In the subtype comparisons, nominal pathway-level patterns involved carbon metabolism, thyroid hormone-related pathways, and pentose and glucuronate interconversions for IFH vs. NGT (Figure 5A and Table S9); amino acid, purine, and nucleotide-related pathways for IPH vs. NGT (Figure 5B and Table S10); and several amino acid-related pathways for CH vs. NGT (Figure 5C and Table S11). These findings are presented solely for exploratory and hypothesis-generating purposes.

Exploratory comparison of the nominal pathway-level patterns across GDM subtypes suggested differences in the pathways represented by the candidate feature sets. In the IFH vs. NGT comparison, these included pathways related to ABC transporters, pyruvate metabolism, oxidative phosphorylation, and thyroid hormone signaling. In the IPH vs. NGT comparison, nominal pathway-level signals included 2-oxocarboxylic acid metabolism, nitrogen metabolism, and the HIF-1 signaling pathway. In the CH vs. NGT comparison, candidate features mapped to pathways involving glycine, serine and threonine metabolism, cysteine and methionine metabolism, and D-amino acid metabolism, among others (Table 3). These comparisons were descriptive and were not intended to establish statistically significant or subtype-specific pathway differences.

## 4. Discussion

In this nested case–control study, we explored first-trimester plasma metabolic profiles among women who subsequently developed GDM and its glycemic subtypes. After correction for multiple testing, no individual metabolic feature remained statistically significant at an FDR threshold of *q* < 0.05. Therefore, the feature-level findings of this study should not be interpreted as confirmed metabolic differences or biomarkers of GDM or its subtypes. Nevertheless, several features showed nominal between-group differences and were retained for exploratory and hypothesis-generating analyses. These nominal candidate features showed variation in chemical-class distributions across glycemic subtypes and mapped to several metabolic pathways. Given the small sample size, high dimensionality of the metabolomic dataset, and absence of FDR-significant individual features, these patterns should be regarded as preliminary observations that may help prioritize hypotheses for future targeted studies rather than as evidence of established subtype-specific metabolic signatures.

Reactive carbonyl stress and the formation of advanced glycation end products (AGEs) have been implicated in diabetes-related metabolic dysfunction [21,22]. In the present exploratory analysis, organosulfur-related annotations were observed among the nominal candidate features in the IFH vs. NGT comparison, whereas alkaloid-related annotations were observed among those in the CH vs. NGT comparison. Previous experimental studies have reported anti-glycation effects of some organosulfur compounds [23], while certain alkaloids have shown glucose-related biological activities, including α-glucosidase inhibition and attenuation of post-load glucose excursions in experimental models [24]. Other experimental studies have suggested that alkaloid-containing preparations may influence glucose metabolism through pathways related to gut microbiota, inflammatory signaling, and hepatic gluconeogenesis [25,26,27]. However, these previous findings provide biological context rather than validation of the subtype-related signals observed in the present study. Importantly, no individual metabolic feature remained statistically significant after FDR correction, and the metabolite identities in our study were based predominantly on database annotation. Therefore, the observed chemical-class patterns should be considered exploratory and hypothesis-generating.

Other nominal candidate features were annotated to chemical classes including benzenoids and lipids and lipid-like molecules. Previous studies have investigated benzoic-acid-derived compounds for their potential glucokinase-activating activity [28], and alterations in lipid and glycerophospholipid metabolism have been reported in women with GDM [29]. Experimental evidence has also shown that lysophosphatidylcholine species such as LPC (16:0) may influence glucose uptake through GLUT4-related mechanisms in adipocytes [30]. These findings provide a possible biological context for the broader chemical classes represented among our exploratory candidate features. Nevertheless, they should not be interpreted as confirmation that the specific putatively annotated features identified in this study are involved in the pathogenesis of particular GDM subtypes. Further validation using targeted metabolomics, MS/MS confirmation, authentic standards, and larger independent cohorts is required.

We further observed several associations between exploratory candidate metabolic features and maternal clinical characteristics that remained significant after FDR correction of the correlation analyses. These included associations with FBG, BMI, and thyroid-related parameters. Several associations were observed between metabolic features and glucose-related or maternal metabolic features. Similar findings have been reported in previous metabolomic studies showing relationships between early pregnancy metabolites and subsequent glucose intolerance [17]. Thyroid hormones may influence glucose and lipid metabolism through effects on hepatic glucose production, adipose tissue function, and insulin sensitivity [31]. However, these results should be interpreted within the exploratory design of the study. The metabolic features entering the correlation analyses were selected from nominal between-group comparisons, and the relatively small sample size and potential residual confounding may affect the stability of the observed associations. Furthermore, correlation does not establish temporal or causal relationships.

To gain further insight into the biological context of the exploratory metabolic signals, pathway analyses were performed to examine whether the nominal candidate features tended to map to related metabolic processes. In the overall GDM vs. NGT comparison, the candidate features were mapped to 69 pathways with nominal enrichment *p* values < 0.05. Among the most strongly represented pathways, 16 candidate features mapped to general metabolic pathways, 6 to biosynthesis of cofactors, 5 to carbon metabolism, 5 to biosynthesis of amino acids, and 4 to purine metabolism. Because these pathway analyses were based on candidate features that did not remain significant after feature-level FDR correction, because several pathways were represented by relatively few features, and because the pathway enrichment *p* values were not adjusted for multiple pathway testing, these findings should be considered exploratory and hypothesis-generating rather than evidence of statistically significant pathway-level alterations.

From a broader clinical perspective, GDM is generally diagnosed at 24–28 weeks of gestation, and there has therefore been substantial interest in identifying early-pregnancy markers associated with subsequent GDM. Previous studies have evaluated maternal characteristics, including age, BMI, medical history, and family history, together with routinely available clinical biomarkers such as fasting blood glucose and glycated hemoglobin, for early GDM risk assessment [32,33]. More recently, metabolomic studies have investigated whether first-trimester metabolites may provide additional information beyond conventional clinical factors [34,35,36]. Some studies have reported improved predictive performance after incorporating selected metabolic markers into traditional risk models [34,35,36]. However, metabolomics-based prediction of GDM remains at an exploratory stage, and differences in study populations, analytical platforms, metabolite identification, and validation strategies have limited the reproducibility and clinical translation of these models. In the present study, the low cross-validated Q^2^ values and the absence of FDR-significant individual metabolic features do not support a predictive interpretation of our data. Accordingly, our pathway findings are intended to generate hypotheses regarding early metabolic alterations associated with subsequent GDM rather than to establish a clinically applicable prediction model.

In enrichment analyses, for subtypes specifically, (1) a comparison between the IFH and NGT groups identified 21 distinct metabolic pathways that were observed in previous study. Among these, the top 5 pathways were ABC transporters (3 CMs), pyruvate metabolism (2 CMs), propanoate metabolism (2 CMs), glycerophospholipid metabolism (2 CMs) and tyrosine metabolism (2 CMs); (2) between IPH and NGT groups, exclusive pathways including metabolic pathways (10 CMs), 2-Oxocarboxylic acid metabolism (2 CMs), nitrogen metabolism (1 CMs), HIF-1 signaling pathway (1 CMs), proximal tubule bicarbonate reclamation (1 CMs); and (3) 11 metabolic pathways between CH and NGT groups were identified, including glycine, serine and threonine metabolism (3 CMs), cysteine and methionine metabolism (3 CMs), D-Amino acid metabolism (3 CMs), aminoacyl-tRNA biosynthesis (3 CMs), protein digestion and absorption (3 CMs), etc.

For IFH, exploratory pathway mapping included ABC transporters, pyruvate metabolism, glycerophospholipid metabolism, and several amino acid-related pathways. These pathways have previously been implicated in glucose homeostasis and insulin sensitivity [37,38,39,40]. In particular, malic acid and succinate mapped to pyruvate metabolism, a pathway involved in glucose regulation. Experimental evidence has shown that PDK4 deficiency can improve glucose tolerance and reduce blood glucose levels in obese mice [41], although the relevance of these findings to human pregnancy remains uncertain. In addition, several candidate features mapped to thyroid hormone-related pathways. Thyroid hormones are involved in glucose and lipid metabolism [42], and epidemiological studies have reported associations between free thyroxine and metabolic parameters such as fasting glucose and triglycerides [43]. Given the differences in thyroid function parameters observed between groups, these findings may suggest a possible relationship between thyroid status and the exploratory metabolic patterns observed in IFH; however, this hypothesis requires confirmation in larger and independent studies. For IPH, several metabolic features were mapped to pathways related to amino acid metabolism and 2-oxocarboxylic acid metabolism. Alpha-ketoglutarate and related metabolites have been implicated in glucose metabolism through regulation of hepatic gluconeogenesis and cellular metabolic signaling [44]. Further studies are needed to determine whether these metabolic alterations are involved in the development of isolated postprandial hyperglycemia. For CH, which represents combined fasting and post-load hyperglycemia, metabolic features were enriched in pathways involving glycine, serine, threonine, cysteine, and methionine metabolism. Previous studies have reported alterations in amino acid metabolism among women with GDM [45]. These nominal pathway-level patterns may provide hypotheses regarding metabolic processes potentially relevant to combined hyperglycemia; however, they should not be interpreted as evidence of confirmed pathway alterations and require independent validation.

Compared with previous metabolomic studies of GDM, our study has several strengths. First, plasma samples were collected during the first trimester, before clinical diagnosis of GDM, allowing exploration of early metabolic differences associated with later GDM subtypes. Second, classification of GDM according to fasting and post-load glucose patterns enabled investigation of metabolic heterogeneity within GDM.

Several limitations of this study should be acknowledged. First, the sample size was relatively small, particularly for the subtype analyses, in which each group included only 30 participants. In the context of more than 20,000 metabolomic features, this substantially limited statistical power after correction for multiple comparisons. Importantly, no individual metabolic feature remained statistically significant after Benjamini–Hochberg FDR correction. Accordingly, the nominal feature-level associations reported in this study should be interpreted exclusively as exploratory candidate signals rather than confirmed metabolic alterations. Second, although OPLS-DA was used to visualize group-related metabolic patterns, the low cross-validated Q^2^ values did not support reliable discrimination between groups. OPLS-DA was therefore interpreted only as an exploratory visualization approach in the present dataset, and VIP values were used solely for exploratory feature ranking rather than as evidence of predictive performance. Third, metabolite identification was based predominantly on database annotation, and most annotations were not confirmed using authentic standards. Some putatively annotated compounds may therefore represent exogenous exposures, contaminants, or annotation artefacts. Future studies using MS/MS confirmation, authentic standards, solvent blanks, and targeted metabolomic approaches are required to verify these identities. Fourth, the same NGT reference group was used in the IFH, IPH, and CH comparisons. The subtype analyses are therefore statistically dependent and should not be interpreted as independent replication analyses. Fifth, pathway enrichment analyses were based on exploratory candidate feature sets, and several pathways contained only one or a few mapped features. In addition, the pathway enrichment *p* values were nominal and were not adjusted for multiple pathway testing. Consequently, the pathway-level findings may be unstable and should be interpreted solely as exploratory and hypothesis-generating rather than as statistically significant or subtype-specific biological mechanisms. Sixth, although the available historical analytical report documented 14 pooled QC injections, QC clustering and correlation assessments, internal-standard response stability, and the use of blank samples, some details of the original analytical sequence could not be fully reconstructed. In particular, the available records did not specify a prespecified fixed QC injection frequency, the procedure used for sample run-order randomization, or whether explicit batch-effect assessment and batch-correction procedures were applied. These remaining uncertainties limit a complete retrospective assessment of potential analytical drift and batch-related variation. Seventh, residual confounding cannot be excluded. Gestational age at first-trimester blood collection was not included in the propensity-score model or metabolomic adjustment. In addition, although blood samples were collected after at least 8 h of fasting and generally during the morning, exact collection times varied according to participant attendance and were not adjusted for in the analyses. These factors may contribute to variability in metabolomic profiles. Larger prospective studies with independent validation cohorts, standardized pre-analytical and analytical procedures, targeted metabolomics, comprehensive QC and batch documentation, and rigorous statistical control are required to determine whether any of the candidate metabolic signals identified here are reproducible.

## 5. Conclusions

In conclusion, this exploratory study characterized first-trimester plasma metabolic profiles among women who subsequently developed GDM and its glycemic subtypes. No individual metabolic feature remained statistically significant after correction for multiple testing. Nevertheless, several nominal candidate features and exploratory pathway-level patterns were observed across the GDM subtype comparisons. These findings should be regarded as hypothesis-generating rather than as validated metabolic biomarkers or subtype-specific signatures. Confirmation using targeted metabolomics, larger independent prospective cohorts, and rigorous metabolite identification is required before the biological or clinical relevance of these candidate signals can be established.

## Figures and Tables

**Table 1:** (**a**) Demographic characteristics in pregnant women with GDM. (**b**) Demographic characteristics in pregnant women with GDM subtypes.

(**a**) Demographic characteristics in pregnant women with GDM					
	**Total**	**Non-GDM**		**GDM**	***p***
	**n = 120**	**n = 30**		**n = 90**	
Age, mean (SD), year	30.15 (3.87)	30.50 (3.80)		30.03 (3.91)	0.57
Education, n (%)					
≤Middle school	13 (10.83)	2 (6.67)		11 (12.22)	0.092
High school	22 (18.33)	5 (16.67)		17 (18.89)	
College or above	57 (47.50)	11 (36.67)		46 (51.11)	
Unknown	28 (23.33)	12 (40.00)		16 (17.78)	
Parity, n (%)					
Primipara	65 (54.17)	12 (40.00)		53 (58.89)	0.143
Multipara	43 (35.83)	13 (43.33)		30 (33.33)	
Unknown	12 (10.00)	5 (16.67)		7 (7.78)	
SBP, mean (SD), mmHg	105.05 (10.48)	106.47 (10.54)		104.58 (10.47)	0.395
DBP, mean (SD), mmHg	69.57 (6.93)	70.07 (6.74)		69.40 (7.02)	0.65
BMI, mean (SD), kg/m^2^	22.16 (2.78)	22.14 (2.48)		22.16 (2.89)	0.972
BMI grouping, n (%)					
Underweight	12 (10.00)	3 (10.00)		9 (10.00)	0.624
Normal	82 (68.33)	23 (76.67)		59 (65.56)	
Overweight	21 (17.50)	3 (10.00)		18 (20.00)	
Obese	5 (4.17)	1 (3.33)		4 (4.44)	
Smoking, n (%)	0 (0.00)	0 (0.00)		0 (0.00)	/
Alcohol drinking, n (%)	0 (0.00)	0 (0.00)		0 (0.00)	/
Thyroid hormones in 1st trimester					
FT4, median (IQR), pmol/L	10.59 [9.89, 12.28]	11.55 [10.60, 14.42]		10.39 [9.75, 11.61]	**0.001**
FT3, median (IQR), pmol/L	4.94 [4.57, 5.26]	4.95 [4.53, 5.10]		4.92 [4.58, 5.29]	0.411
TSH, median (IQR), mIU/L	1.05 [0.58, 1.75]	0.80 [0.52, 1.21]		1.24 [0.62, 1.93]	0.054
FT3/FT4	0.44 [0.40, 0.50]	0.41 [0.33, 0.46]		0.46 [0.41, 0.51]	**0.001**
TT4RI	12.29 [6.51, 19.12]	10.48 [5.86, 15.40]		12.72 [6.55, 20.07]	0.237
TSHI	1.63 [0.95, 2.06]	1.60 [0.98, 1.91]		1.63 [0.95, 2.07]	0.676
TFQI	−0.07 [−0.32, 0.09]	0.01 [−0.20, 0.11]		−0.11 [−0.36, 0.08]	0.111
TPOAb positive, n (%)	5 (4.17)	2 (6.67)		3 (3.33)	0.792
TgAb positive, n (%)	2 (1.67)	2 (6.67)		0 (0.00)	0.1
Glucose levels in OGTT, median (IQR)					
GLU	5.08 [4.49, 5.30]	4.50 [4.24, 4.64]		5.20 [4.73, 5.41]	<0.001
PBG1H	9.22 [7.98, 10.37]	7.97 [6.89, 8.94]		9.94 [8.78, 10.55]	<0.001
PBG2H	8.10 [7.14, 8.79]	7.18 [6.26, 7.80]		8.52 [7.58, 8.96]	<0.001
Weight gain, mean (SD), kg	5.55 (2.40)	4.97 (2.47)		5.74 (2.36)	0.125
(**b**) Demographic characteristics in pregnant women with GDM subtypes.					
	**Non-GDM**	**IFH**	**IPH**	**CH**	***p***
	**n = 30**	**n = 30**	**n = 30**	**n = 30**	
Age, mean (SD), year	30.50 (3.80)	29.97 (4.44)	30.20 (3.45)	29.93 (3.90)	0.94
Education, n (%)					
≤Middle school	2 (6.67)	3 (10.00)	5 (16.67)	3 (10.00)	0.328
High school	5 (16.67)	6 (20.00)	3 (10.00)	8 (26.67)	
College or above	11 (36.67)	16 (53.33)	15 (50.00)	15 (50.00)	
Unknown	12 (40.00)	5 (16.67)	7 (23.33)	4 (13.33)	
Parity, n (%)					
Primipara	12 (40.00)	18 (60.00)	17 (56.67)	18 (60.00)	0.287
Multipara	13 (43.33)	12 (40.00)	9 (30.00)	9 (30.00)	
Unknown	5 (16.67)	0 (0.00)	4 (13.33)	3 (10.00)	
SBP, mean (SD), mmHg	106.47 (10.54)	102.73 (8.12)	103.87 (8.34)	107.13 (13.80)	0.313
DBP, mean (SD), mmHg	70.07 (6.74)	67.53 (7.06)	68.87 (5.77)	71.80 (7.62)	0.102
BMI, mean (SD), kg/m^2^	22.14 (2.48)	21.80 (3.05)	21.96 (2.57)	22.72 (3.02)	0.603
BMI grouping, n (%)					
Underweight	3 (10.00)	4 (13.33)	4 (13.33)	1 (3.33)	0.63
Normal	23 (76.67)	20 (66.67)	19 (63.33)	20 (66.67)	
Overweight	3 (10.00)	4 (13.33)	7 (23.33)	7 (23.33)	
Obese	1 (3.33)	2 (6.67)	0 (0.00)	2 (6.67)	
Smoking, n (%)	0 (0.00)	0 (0.00)	0 (0.00)	0 (0.00)	/
Alcohol drinking, n (%)	0 (0.00)	0 (0.00)	0 (0.00)	0 (0.00)	/
Thyroid hormones in 1st trimester					
FT4, median (IQR), pmol/L	11.55 [10.60, 14.42]	10.37 [9.88, 12.25]	10.52 [9.75, 12.03]	10.38 [9.58, 10.90]	**0.007**
FT3, median (IQR), pmol/L	4.95 [4.53, 5.10]	5.09 [4.77, 5.29]	4.89 [4.58, 5.29]	4.74 [4.57, 5.19]	0.465
TSH, median (IQR), mIU/L	0.80 [0.52, 1.21]	1.01 [0.45, 2.09]	1.32 [0.81, 1.71]	1.23 [0.63, 1.91]	0.236
FT3/FT4	0.41 [0.33, 0.46]	0.46 [0.39, 0.51]	0.46 [0.41, 0.51]	0.46 [0.42, 0.51]	**0.013**
TT4RI	10.48 [5.86, 15.40]	9.72 [4.92, 21.18]	14.21 [7.94, 17.04]	12.45 [6.55, 19.28]	0.625
TSHI	1.60 [0.98, 1.91]	1.32 [0.65, 2.13]	1.69 [1.10, 2.01]	1.58 [0.93, 2.06]	0.893
TFQI	0.01 [−0.20, 0.11]	−0.12 [−0.40, 0.08]	−0.07 [−0.28, 0.09]	−0.16 [−0.34, 0.07]	0.392
TPOAb positive, n (%)	2 (6.67)	1 (3.33)	1 (3.33)	1 (3.33)	0.89
TgAb positive, n (%)	2 (6.67)	0 (0.00)	0 (0.00)	0 (0.00)	0.107
**Glucose levels in OGTT, median (IQR)**					
GLU	4.50 [4.24, 4.64]	5.32 [5.24, 5.48]	4.44 [4.29, 4.72]	5.30 [5.18, 5.49]	**<0.001**
PBG1H	7.97 [6.89, 8.94]	8.03 [7.47, 9.07]	10.04 [9.16, 10.61]	10.46 [10.04, 11.33]	**<0.001**
PBG2H	7.18 [6.26, 7.80]	7.24 [6.67, 7.84]	8.75 [8.53, 9.16]	8.84 [8.55, 9.29]	**<0.001**
**Weight gain, mean (SD), kg**	4.97 (2.47)	6.58 (2.81)	5.56 (2.10)	5.09 (1.91)	**0.036**

**Reference values:** FT3, 3.10–6.80 pmol/L; FT4, 12.00–22.00 pmol/L; TSH, 0.27–4.20 μIU/mL. TPOAb positivity: >34.0 IU/mL; TgAb positivity: >115 IU/mL. Bold values indicate statistical significance (*p* < 0.05).

**Table 2:** Maternal FBG, thyroid hormones, and anthropometric traits correlate with candidate metabolic features between subtyped GDM and NGT groups (r > 0.3 and *q* < 0.05).

Variables	Positive Correlation	Negative Correlation
IFH vs. NGT		
Education	Anticopalic acid//Arachidonic acid (AA)//Cyclocytidine//Methylmalonic acid//Succinate	Hydrastinine//4-tert-Butyl-N-(4-nitrophenyl)benzamide
Parity	/	[(7-Oxo-7H-benzo[de]anthracen-3-yl)sulfanyl]acetic acid //4′,5,7-Trihydroxy 3,3′,6,8-tetramethoxyflavone
BMI	/	Vamidothion
FT4	16-Hydroxypalmitic acid//2-Hydroxypalmitic acid//Anticopalic acid//Arachidonic acid (AA)//LPC(13:0)//VERAPAMIL	
FT3/FT4 ratio	/	Anticopalic acid//Arachidonic acid (AA)//Malic acid
Weight_gain	Carbamoyl_phosphate//Vamidothion	/
IPH vs. NGT		
Education	/	1-(Cyclopentylcarbonyl)-4-piperidinamine//1,3-Diaminopropane-N,N,N′,N′-tetraacetic acid//DL-Norvaline//M351T239_1//Mavorixafor//Monobutyl phthalate//PC(18:3(6Z,9Z,12Z)/18:0)
FT4	/	1-(Cyclopentylcarbonyl)-4-piperidinamine//Monobutyl phthalate
FT3/FT4 ratio	Monobutyl phthalate	/
CH vs. NGT		
Education	/	1-(3-Cyclohexen-1-ylmethyl)-4-piperidinamine//1-(Cyclopentylcarbonyl)-4-piperidinamine//1,3-Diaminopropane-N,N,N′,N′-tetraacetic acid//1,3,7-Trimethyluric acid//2-(1-Pyrrolidinyl)-3-pentanone//2-(4-Bromophenyl)-4(3H)-quinazolinone//2-Methoxy-4-(4-morpholinylcarbothioyl)phenol//2-Methylpiperidine//2,6-Di-tert-butyl-4-(4-morpholinylmethyl)phenol//3-[5-[Acetyl(hydroxy)amino]pentylcarbamoyl]-5-[3-[acetyl(hydroxy)amino]propylamino]-3-hydroxy-5-oxopentanoic acid//3-Iodothyronamine//6-Deoxyfagomine//7-Methyl-3,4-dihydro-2H-pyrido [1,2-a]pyrimidin-3-ol//a-Santal-10-en-12-oic acid//DL-Norvaline//Florfenicol//M351T239_1//Monobutyl phthalate//N-(4-Piperidinyl)cyclohexanecarboxamide//N-Methyl-N-(methylsulfonyl)glycine//N,N-Diethyl-2-aminoethanol//Nicotine
BMI	2-Aminonicotinic acid//4-Nitroaniline//Nicotinamide N-oxide//PC(38:3)//Threonine	/
FBG	3-[5-[Acetyl(hydroxy)amino]pentylcarbamoyl]-5-[3-[acetyl(hydroxy)amino]propylamino]-3-hydroxy-5-oxopentanoic acid	/
TSH	2,6-Di-tert-butyl-4-(4-morpholinylmethyl)phenol//3-Iodothyronamine//Florfenicol	/
FT4	/	1-(Cyclopentylcarbonyl)-4-piperidinamine//Monobutyl phthalate
FT3	O-Succinyl-L-homoserine//Oxypurinol//Xanthine	/
FT3/FT4 ratio	(1,3-Benzothiazol-2-ylsulfonyl)acetic acid//1-(3-Cyclohexen-1-ylmethyl)-4-piperidinamine//1-(3,4-Dichlorophenyl)-3-phenyl-2-thiourea//1-(Cyclopentylcarbonyl)-4-piperidinamine//1-Palmitoyl-3-oleoyl-sn-glycero-2-phosphoethanolamine//1,3-Diaminopropane-N,N,N′,N′-tetraacetic acid//1,3,7-Trimethyluric acid//2-(1-Pyrrolidinyl)-3-pentanone//2-(4-Bromophenyl)-4(3H)-quinazolinone//2-Aminonicotinic acid//2-Methoxy-4-(4-morpholinylcarbothioyl)phenol//2-Methylpiperidine//2,6-Di-tert-butyl-4-(4-morpholinylmethyl)phenol//3-[5-[Acetyl(hydroxy)amino]pentylcarbamoyl]-5-[3-[acetyl(hydroxy)amino]propylamino]-3-hydroxy-5-oxopentanoic acid//3-Iodothyronamine//4-Nitroaniline//6-Deoxyfagomine//7-Methyl-3,4-dihydro-2H-pyrido [1,2-a]pyrimidin-3-ol//a-Santal-10-en-12-oic acid//DL-Norvaline//Florfenicol//Glucuronic acid//Monobutyl phthalate//N-(4-Chlorophenyl)-1,3-benzothiazol-2-amine//N-(4-Piperidinyl)cyclohexanecarboxamide//N-Methyl-N-(methylsulfonyl)glycine//N,N-Diethyl-2-aminoethanol//Nicotinamide N-oxide//Nicotine//PC(38:3)//PI(18:0/22:6(4Z,7Z,10Z,13Z,16Z,19Z))	Pyripyropene A

The Spearman correlations between detected metabolites and fasting blood glucose concentrations (FBG), maternal BMI, thyroid hormones, maternal education, parity and weight gain in the entire cohort, with a correlation coefficient greater than 0.3 and a *q* value < 0.05.

**Table 3:** The differences in metabolic pathways among different groups.

IFH vs. NGT	IPH vs. NGT	CH vs. NGT	Count	Metabolic Pathways
TRUE	FALSE	FALSE	21	ABC transporters, Pyruvate metabolism, Propanoate metabolism, Glycerophospholipid metabolism, Tyrosine metabolism, Glucagon signaling pathway, Oxidative phosphorylation, Necroptosis, Platelet activation, Fc epsilon RI signaling pathway, Fc gamma R-mediated phagocytosis, Regulation of lipolysis in adipocytes, GnRH signaling pathway, Oxytocin signaling pathway, Thyroid hormone signaling pathway, Long-term depression, Choline metabolism in cancer, Renal cell carcinoma, Amoebiasis, Leishmaniasis, Autoimmune thyroid disease
FALSE	FALSE	TRUE	11	Glycine, serine and threonine metabolism, Cysteine and methionine metabolism, D-Amino acid metabolism, Aminoacyl-tRNA biosynthesis, Protein digestion and absorption, Ascorbate and aldarate metabolism, beta-Alanine metabolism, Taurine and hypotaurine metabolism, Pantothenate and CoA biosynthesis, Mineral absorption, Nicotine addiction
TRUE	TRUE	TRUE	7	Carbon metabolism, Glyoxylate and dicarboxylate metabolism, Purine metabolism, Pyrimidine metabolism, Alanine, aspartate and glutamate metabolism, Bile secretion, GABAergic synapse
FALSE	TRUE	FALSE	5	Metabolic pathways, 2-Oxocarboxylic acid metabolism, Nitrogen metabolism, HIF-1 signaling pathway, Proximal tubule bicarbonate reclamation

## Data Availability

The data that support the findings of this study are available from the corresponding author on request.

## Supplementary Materials

The following supporting information can be downloaded at: https://www.mdpi.com/article/10.3390/life16101641/s1. Figure S1. PCA model score scatter plot. Figure S2. Distinct separations in metabolic profiles using OPLS-DA. (A): OPLS-DA model of GDM vs. NGT. (B): permutation test of GDM vs. NGT. R2Y describes model fit to the class-response matrix, whereas Q2 represents cross-validated model performance. The low Q2 value did not support reliable discrimination between GDM and NGT. Figure S3. Distribution of chemical classes among exploratory candidate metabolic features. (A–C) Pie charts showing the chemical-class distributions of exploratory candidate metabolic features in the IFH vs. NGT, IPH vs. NGT, and CH vs. NGT comparisons, respectively. Table S1. 107 exploratory candidate metabolic features between GDM and NGT groups. Table S2. 89 exploratory candidate metabolic features between IFH and NGT groups. Table S3. 76 exploratory candidate metabolic features between IPH and NGT groups. Table S4. 123 exploratory candidate metabolic features between CH and NGT groups. Table S5. The clustering and correlations of annotated DEMs in IFH vs. NGTgroups with FBG, maternal BMI, early pregnancy thyroid hormones, weight gain from first trimester to second trimester, education, and parity (n = 89). Table S6. The clustering and correlations of annotated DEMs in IPH vs. NGTgroups with FBG, maternal BMI, early pregnancy thyroid hormones, weight gain from first trimester to second trimester, education, and parity (n = 76). Table S7. The clustering and correlations of annotated DEMs in CH vs. NGTgroups with FBG, maternal BMI, early pregnancy thyroid hormones, weight gain from first trimester to second trimester, education, and parity (n = 123). Table S8. Results of KEGG enrichment analysis of the metabolic pathways between GDM vs. NGT. Table S9. Results of KEGG enrichment analysis of the metabolic pathways between IFH vs. NGT. Table S10. Results of KEGG enrichment analysis of the metabolic pathways between IPH vs. NGT. Table S11. Results of KEGG enrichment analysis of the metabolic pathways between CH vs. NGT.
