# Supplementary material for: Metabolomic Profiling of Gestational Diabetes Mellitus and Its Subtypes Among Chinese Pregnant Women
Source: Life (Basel). 2026 Sep 30;16(10):1641. doi: 10.3390/life16101641 (PMC13706523; doi:10.3390/life16101641)
Supplement: Supplementary file 1 [file life-16-01641-s001.zip › Figure S1.pdf]

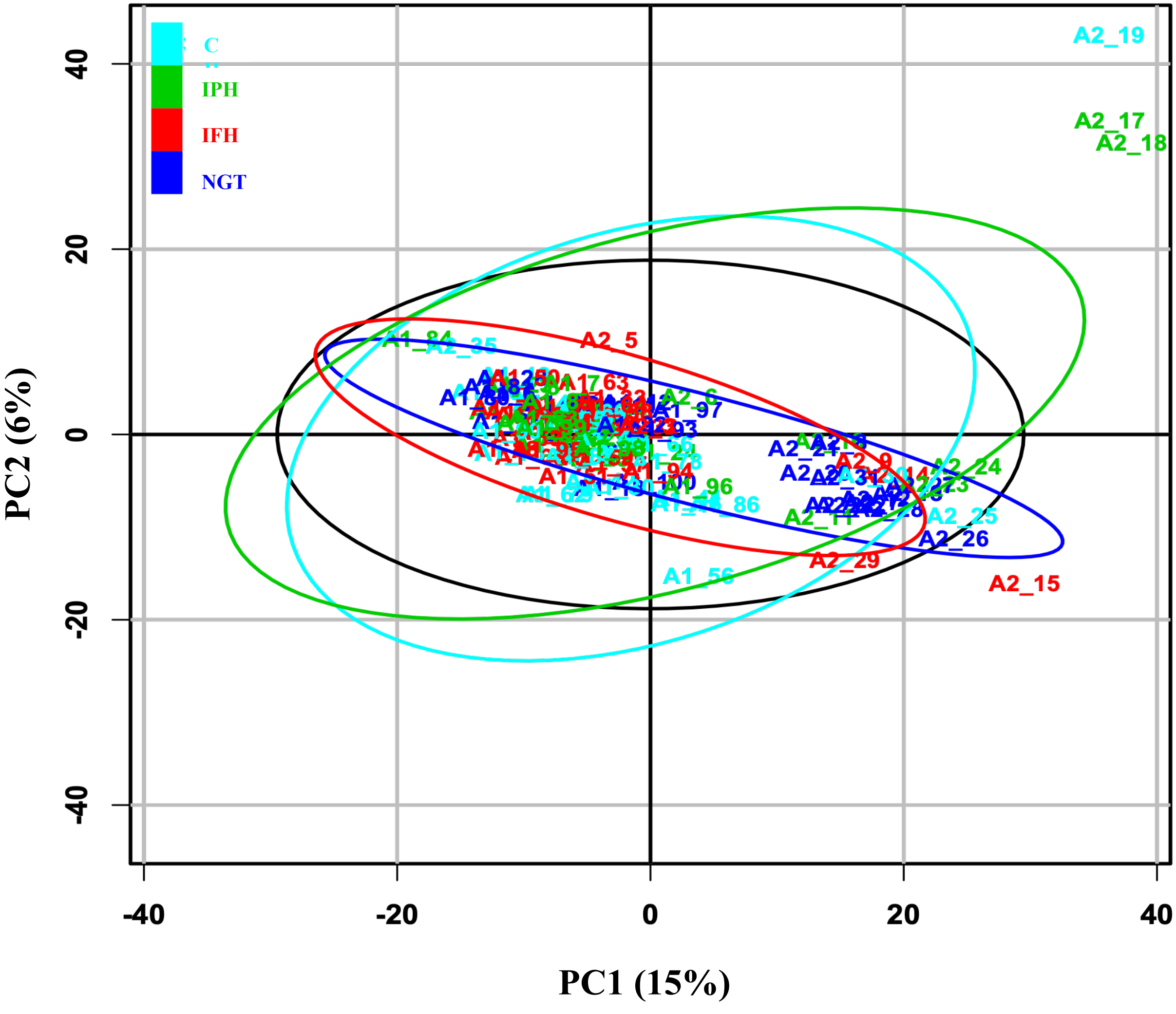
C
IPH
IFH
NGT
PC1 (15%)
PC2 (6%)
-40
-20
0
20
40
A2_19
A2_17
A2_18
A2_5
A2_35
A2_24
A2_25
A2_26
A2_29
A2_15
A1_56
