# Supplementary material for: Metabolomic Profiling of Gestational Diabetes Mellitus and Its Subtypes Among Chinese Pregnant Women
Source: Life (Basel). 2026 Sep 30;16(10):1641. doi: 10.3390/life16101641 (PMC13706523; doi:10.3390/life16101641)
Supplement: Supplementary file 1 [file life-16-01641-s001.zip › Figure S2.pdf]

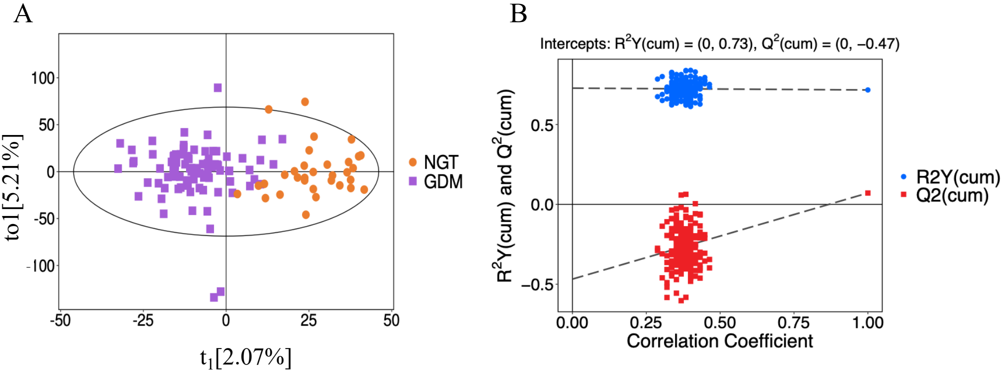

A
100
50
0
-50
-100
-50
-25
0
25
50
to1[5.21%]
t1[2.07%]
NGT
GDM
B
Intercepts: R2Y(cum) = (0, 0.73), Q2(cum) = (0, −0.47)
0.5
0.0
−0.5
0.00
0.25
0.50
0.75
1.00
R2Y(cum) and Q2(cum)
Correlation Coefficient
R2Y(cum)
Q2(cum)
