# Supplementary material for: Metabolomic Profiling of Gestational Diabetes Mellitus and Its Subtypes Among Chinese Pregnant Women
Source: Life (Basel). 2026 Sep 30;16(10):1641. doi: 10.3390/life16101641 (PMC13706523; doi:10.3390/life16101641)
Supplement: Supplementary file 1 [file life-16-01641-s001.zip › Figure S3.pdf]

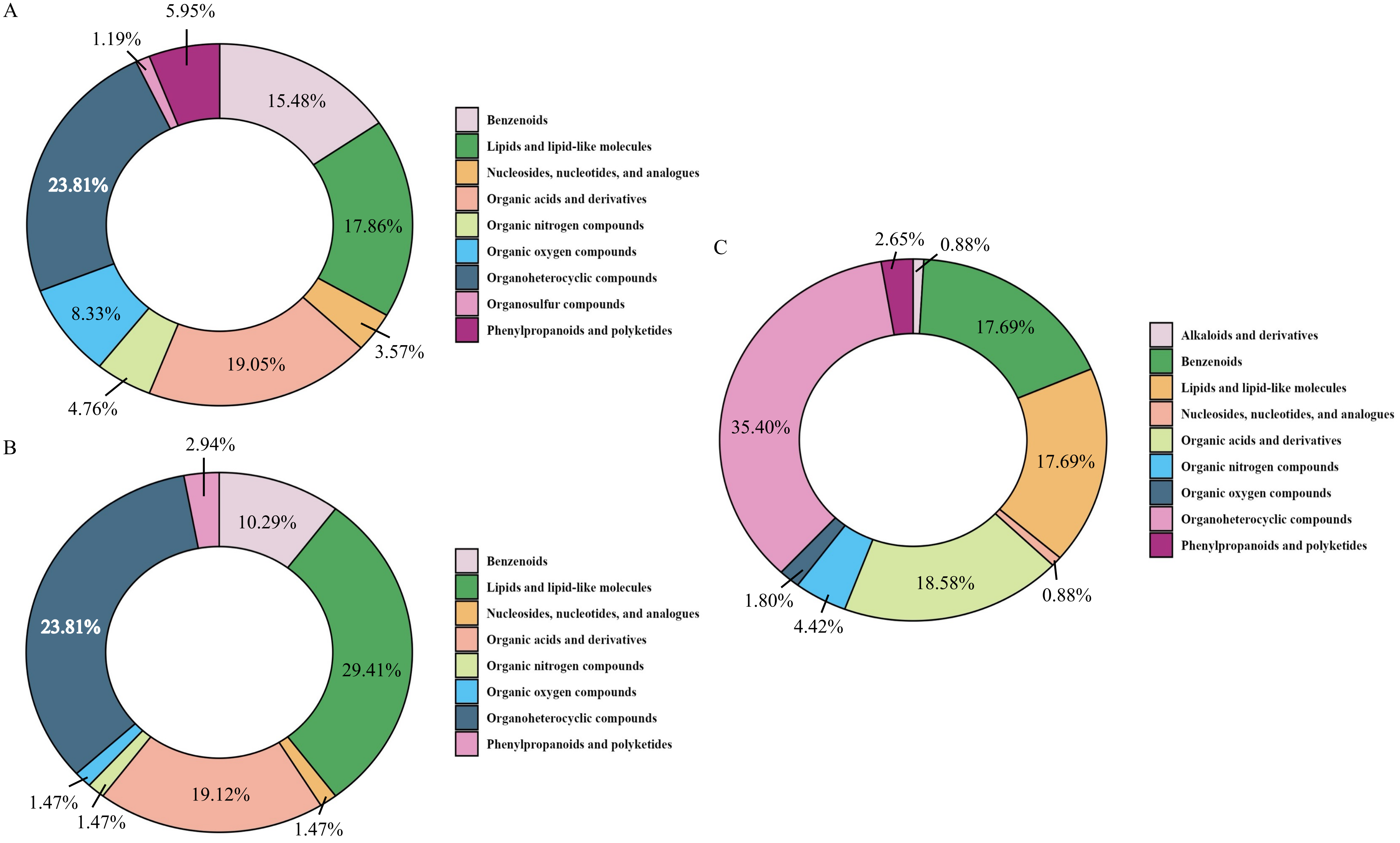
A
5.95%
1.19%
15.48%
23.81%
17.86%
8.33%
3.57%
19.05%
4.76%
Benzenoids
Lipids and lipid-like molecules
Nucleosides, nucleotides, and analogues
Organic acids and derivatives
Organic nitrogen compounds
Organic oxygen compounds
Organoheterocyclic compounds
Organosulfur compounds
Phenylpropanoids and polyketides
B
2.94%
10.29%
23.81%
29.41%
19.12%
1.47%
1.47%
1.47%
Benzenoids
Lipids and lipid-like molecules
Nucleosides, nucleotides, and analogues
Organic acids and derivatives
Organic nitrogen compounds
Organic oxygen compounds
Organoheterocyclic compounds
Phenylpropanoids and polyketides
C
2.65%
0.88%
17.69%
35.40%
17.69%
18.58%
0.88%
1.80%
4.42%
Alkaloids and derivatives
Benzenoids
Lipids and lipid-like molecules
Nucleosides, nucleotides, and analogues
Organic acids and derivatives
Organic nitrogen compounds
Organic oxygen compounds
Organoheterocyclic compounds
Phenylpropanoids and polyketides
